# Real-Time Heartbeat Classification on Distributed Edge Devices: A Performance and Resource Utilization Study

**DOI:** 10.3390/s25196116

**Published:** 2025-10-03

**Authors:** Eko Sakti Pramukantoro, Kasyful Amron, Putri Annisa Kamila, Viera Wardhani

**Affiliations:** 1 Faculty of Computer Science, Universitas Brawijaya, Malang 65145, Indonesia; kasyful@ub.ac.id; 2 Faculty of Medicine, Universitas Brawijaya, Malang 65145, Indonesia; putriannisa@ub.ac.id (P.A.K.); viera_w.fk@ub.ac.id (V.W.)

**Keywords:** heartbeat classification, LSTM, stream processing, Jetson Nano, real-time inference

## Abstract

Early detection is crucial for preventing heart disease. Advances in health technology, particularly wearable devices for automated heartbeat detection and machine learning, can enhance early diagnosis efforts. However, previous studies on heartbeat classification inference systems have primarily relied on batch processing, which introduces delays. To address this limitation, a real-time system utilizing stream processing with a distributed computing architecture is needed for continuous, immediate, and scalable data analysis. Real-time ECG inference is particularly crucial for immediate heartbeat classification, as human heartbeats occur with durations between 0.6 and 1 s, requiring inference times significantly below this threshold for effective real-time processing. This study implements a real-time heartbeat classification inference system using distributed stream processing with LSTM-512, LSTM-256, and FCN models, incorporating RR-interval, morphology, and wavelet features. The system is developed as a distributed web-based application using the Flask framework with distributed backend processing, integrating Polar H10 sensors via Bluetooth and Web Bluetooth API in JavaScript. The implementation consists of a frontend interface, distributed backend services, and coordinated inference processing. The frontend handles sensor pairing and manages real-time streaming for continuous ECG data transmission. The backend processes incoming ECG streams, performing preprocessing and model inference. Performance evaluations demonstrate that LSTM-based heartbeat classification can achieve real-time performance on distributed edge devices by carefully selecting features and models. Wavelet-based features with an LSTM-Sequential architecture deliver optimal results, achieving 99% accuracy with balanced precision-recall metrics and an inference time of 0.12 s—well below the 0.6–1 s heartbeat duration requirement. Resource analysis on Jetson Orin devices reveals that Wavelet-FCN models offer exceptional efficiency with 24.75% CPU usage, minimal GPU utilization (0.34%), and 293 MB memory consumption. The distributed architecture’s dynamic load balancing ensures resilience under varying workloads, enabling effective horizontal scaling.

## 1. Introduction

Cardiovascular diseases, commonly known as heart diseases, are among the leading causes of global mortality, accounting for approximately 31% of all deaths worldwide (WHO, 2024). In 2019 alone, these diseases led to the deaths of 17.9 million people (WHO, 2024). The World Health Organization (WHO) has emphasized that heart disease can be prevented through early detection [1].

Early detection is vital for determining appropriate treatment and medical interventions, which can significantly reduce the risk of serious complications and fatalities. Traditional diagnostic methods often require clinical visits and specialized medical equipment, which may not always be accessible or timely for at-risk patients. Therefore, advancements in healthcare technology have become increasingly crucial in supporting early diagnosis efforts [2,3].

A promising approach involves automating heartbeat detection using wearable devices combined with machine learning algorithms [4,5,6]. These technologies can continuously monitor heart activity, analyze physiological patterns, and provide real-time insights that help healthcare professionals diagnose heart disease at an early stage. By leveraging artificial intelligence (AI) and real-time data processing, such systems can enhance diagnostic accuracy, lessen the burden on medical practitioners, and potentially save lives through timely intervention.

Real-time electrocardiogram (ECG) inference using stream processing is crucial for immediate heartbeat classification. Human heartbeats typically occur within durations of 0.6 to 1 s. Extensive studies involving over 66,000 participants in the Health eHeart Study found that the mean real-world heart rate (HR) is 79.1 beats per minute (bpm) ± 14.5, which translates to approximately 0.758 s per beat. Normal resting HR values for adults generally range from 60 to 90 bpm, corresponding to beat durations of 1 s to approximately 0.667 s. The American Heart Association defines a normal sinus HR as 60–100 bpm, with 100 bpm equating to 0.6 s per beat [7]. This temporal constraint necessitates processing systems capable of detecting and classifying heartbeats within these sub-second intervals, enabling accurate real-time monitoring.

One wearable device that supports the early detection of heart conditions is the Polar H10 [8]. The Polar H10 is a high-precision wearable device designed for recording ECG data, capable of continuous operation for 12 to 14 days. It is widely used by endurance athletes, such as cyclists and marathon runners, due to its exceptional accuracy, even during intense physical activities like running, cycling, and weightlifting. Unlike traditional Holter monitors, which are costly and require clinical setup, the Polar H10 provides a cost-effective and portable alternative for continuous heart monitoring [9]. Additionally, the Polar H10 offers seamless connectivity, supporting Bluetooth Low Energy (BLE) for easy integration with various devices, including smartphones, smartwatches, and edge computing platforms. This wireless capability enhances its usability in real-world applications, allowing for real-time data transmission for further processing.

However, while the Polar H10 excels in ECG data acquisition, it lacks built-in capabilities for real-time ECG interpretation. To maximize its potential for early detection, it must be paired with advanced computing and AI solutions. Previous studies have demonstrated the effectiveness of CNN-based and LSTM-based models for automatic heartbeat detection [10,11]. These models are particularly suitable for long-term monitoring with wearable devices, providing a cost-effective alternative to traditional Holter monitors [9]. Regarding the computational cost, LSTM-FCN models are the most efficient option for low-latency scenarios compared to CNN-based models. Specifically, the R-R interval (RRI)-based LSTM-FCN achieved the fastest inference time, while the morphology-based LSTM-FCN delivered the highest classification accuracy [12]. Thus, LSTM is particularly well-suited for making real-time predictions in automatic heartbeat classification.

In this study, we introduce a comprehensive real-time ECG inference system that utilizes advanced stream processing techniques. This system integrates the Polar H10 sensor via Bluetooth Low Energy (BLE) with a high-performance edge computing node. By combining the Polar H10 with an AI-powered inference mechanism, we can achieve continuous monitoring and real-time heartbeat classification.

Our proposed architecture addresses the significant limitations of traditional batch processing systems by enabling continuous ECG analysis, thereby eliminating data accumulation delays. The ECG data from the sensor is transmitted to a web frontend via the Web Bluetooth API and is processed using optimized deep learning models, including LSTM-512, LSTM-256, and Fully Connected Networks (FCN), which are delivered as distributed web services.

Real-time capability is essential for immediate anomaly detection and timely intervention, both of which are crucial for patient safety. The system classifies each heartbeat in real time, based on standard categories recommended by the Association for the Advancement of Medical Instrumentation (AAMI). These categories include: N (normal beats), S (supraventricular ectopic beats), V (ventricular ectopic beats), F (fusion beats), and Q (unclassifiable beats) [13]. This classification ensures clinical relevance and interoperability with existing diagnostic frameworks.

Previous studies have demonstrated that batch-based post-analysis systems using Polar H10 recordings suffer from increasing inference delays as the data length increases. For example, delays of 0.926 s for 10 s of data, 2.757 s for 60 s, and up to 14.871 s for 600 s highlight a significant latency bottleneck in non-streaming architectures [12]. By leveraging distributed stream processing, our system overcomes these challenges, enabling low-latency, scalable, and responsive ECG analysis. This capability lays a strong foundation for real-time health monitoring applications.

To ensure reliability, we deploy the system using a RESTful web service architecture hosted on an NVIDIA Jetson Orin Nano device, known for its high-performance edge AI capabilities and energy efficiency. Although the Jetson platform exceeds the power budget typically allocated for wearable devices, its centralized deployment permits real-time inference for multiple patients simultaneously without relying on cloud services. This makes it particularly suitable for hospital-based monitoring scenarios. The use of platform-independent REST APIs promotes interoperability and seamless integration with existing healthcare systems.

In summary, our proposed system presents a unified, end-to-end architecture that supports continuous ECG acquisition, low-latency AI-based classification, and scalable deployment for real-time cardiac monitoring. The following sections will elaborate on the system design, implementation, and empirical evaluation, validating its performance and applicability.

## 2. A State of the Art

Heart disease, particularly cardiovascular disease (CVD), remains the leading cause of death globally, highlighting the need for early detection and continuous monitoring of cardiac activity. Several studies have been conducted to develop real-time ECG monitoring and classification systems using deep learning and IoT-based technologies.

A study by Liu et al. [14] introduced a real-time arrhythmia monitoring system utilizing a quantized 1D CNN model trained on the MIT-BIH Arrhythmia Database. Their approach aimed to address the inefficiencies of manual ECG interpretation, which typically requires 24 to 48 h for recording and expert analysis. Unlike conventional methods, their system directly processed raw ECG signals without preprocessing and was trained on an Nvidia Tesla P100 GPU for over 100 epochs. The proposed model was deployed on edge devices, including Raspberry Pi and smartphones, with inference times of 4.76 ms on Raspberry Pi and 7.65 ms on smartphones. Additionally, the system included a real-time ECG visualization feature, assisting healthcare professionals in validating classification results. While the study successfully deployed an efficient deep learning model for arrhythmia detection, it lacked detailed specifications on the wearable device used for ECG data collection. This research inspired our study, particularly in integrating ECG chart visualization, which aids healthcare professionals in performing cross-checks and real-time analysis.

In a separate study, Canon et al. [15] focused on ECG monitoring for individuals in remote and rural areas with limited access to hospitals and healthcare professionals. Their research projected an increase in CVD-related deaths to 23.3 million by 2030, emphasizing the importance of accessible, real-time cardiac monitoring. To address this challenge, they proposed an IoT-based ambulatory ECG monitoring system that leverages low-power IoT technology. The core of their system was the DATU device, equipped with three electrodes for ECG acquisition. Data collected by these electrodes was processed using an Analog Front-End (AFE) before being transmitted to a smartphone for real-time streaming and heart rate calculation. The smartphone then stored the processed data in Google Cloud Firebase, enabling doctors to remotely access and analyze patient ECG readings. The system also included bradycardia and tachycardia detection, with automated alerts sent to both doctors and patients. The system achieved a latency of 0.75 s, making it suitable for real-time streaming, and utilized 50 Hz frequency filtering to improve ECG signal clarity. The integration of cloud computing and mobile applications significantly improved accessibility, particularly for underserved regions. This research provided valuable insights into wearable device design and low-power system implementation, two crucial elements for developing efficient real-time ECG monitoring solutions.

Another notable study, conducted by Hizem et al. [16], addressed the challenges of manual ECG interpretation, particularly the limited access to trained doctors for ECG monitoring. Their solution involved an IoT-based wireless ECG monitoring system that could transmit ECG data remotely. The system utilized an Arduino ECG AD8232 sensor for data acquisition, with a Raspberry Pi processing the signal before transmitting it to a cloud-based IoT platform via Wi-Fi. This allowed real-time ECG visualization and storage for further analysis. Compared to Bluetooth-based systems, Wi-Fi provided a wider communication range, making the system more adaptable for telemedicine applications. Additionally, the system incorporated an anomaly detection algorithm that automatically triggered email alerts for both patients and doctors when abnormalities in ECG readings were detected. To evaluate accuracy, the device was tested against a standard 12-lead ECG system, analyzing key parameters, including the RR interval, PR interval, QT interval, and QRS complex. The results indicated an overall accuracy of 80%, with minor deviations of less than 10% for RR, QT, and QRS intervals. However, the PR interval exhibited a larger discrepancy of approximately 20%, suggesting room for improvement in precision. This study demonstrated the feasibility of cloud-integrated ECG monitoring with anomaly detection, inspiring us to integrate similar real-time analysis capabilities into our system.

Recent advancements have also explored deep learning-based ECG classification models deployed on edge computing platforms. Researchers have proposed hybrid deep learning architectures, such as CNN-LSTM models, which enhance feature extraction and classification accuracy [17]. Additionally, lightweight LSTM models have been developed specifically for real-time ECG monitoring on edge devices, optimizing both accuracy and computational efficiency [18]. Novel approaches, such as the Mamba-RAYOLO model, integrate advanced modules for real-time ECG image classification, ensuring efficient inference and feature extraction for edge computing applications [19]. Furthermore, tiny matched filter-based CNNs have been proposed to optimize inter-patient ECG classification, emphasizing the importance of lightweight architectures for real-time applications [20].

These studies collectively highlight significant advancements in real-time ECG monitoring and arrhythmia detection using deep learning, the Internet of Things (IoT), and wearable devices. However, most existing solutions either rely on batch processing, suffer from limited computational efficiency on edge devices, or lack detailed specifications on sensor integration. Our study builds upon these advancements by deploying LSTM-based real-time heartbeat classification on the Jetson Orin Nano, a high-performance AI edge device. Unlike previous works, our approach prioritizes stream processing, ensuring minimal latency and continuous ECG monitoring. Additionally, we utilize the Polar H10 as the ECG sensor, leveraging the Web Bluetooth API for seamless integration with a Flask-based web interface. The system is designed to provide real-time inference visualization, optimizing both response time and resource efficiency for practical deployment in healthcare applications.

## 3. Proposed Inference System

**Figure 1:**
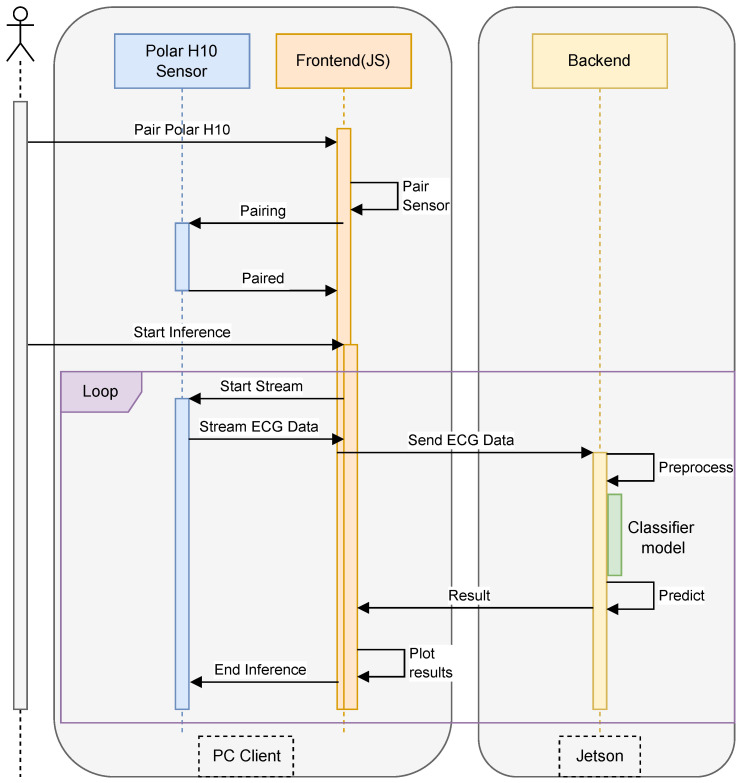
Our inference architecture.

The proposed system, as shown in Figure 1 is designed to enable real-time ECG data inference from the Polar H10 sensor using an edge computing approach with the NVIDIA Jetson. The system architecture comprises three main components: the Polar H10 sensor for ECG data acquisition, a web-based interface for user interaction, and a backend server running on Jetson, which is responsible for data processing and inference. The implementation follows a client–server model, where the front-end web application communicates with the backend using JSON-based messaging.

The system workflow begins with the user initiating the pairing process between the web browser and the Polar H10 sensor via Bluetooth Low Energy (BLE). Once connected, the sensor continuously streams ECG data, which is captured by the front-end and transmitted to the backend via HTTP requests in JSON format. The backend is implemented using Flask, a lightweight web framework that handles data reception, preprocessing, and inference execution. Upon receiving raw ECG signals, the backend applies signal processing techniques such as filtering and normalization to remove noise and enhance feature extraction. Subsequently, the preprocessed data is passed to a deep learning-based inference model, optimized for execution on the Jetson’s GPU, enabling low-latency predictions for stress detection or heart rate classification.

The results of the inference process are then packaged in JSON format and sent back to the web browser, where they are displayed to the user in real-time. The backend is designed as a RESTful API, ensuring seamless integration with various front-end clients while maintaining scalability and flexibility. To optimize performance, the Flask server utilizes asynchronous processing to handle multiple data streams efficiently, thereby preventing bottlenecks during high-frequency ECG data transmission. The integration of Flask, JSON-based communication, and Jetson’s accelerated computing capabilities ensures an efficient, real-time ECG inference system, making it suitable for continuous health monitoring applications.

This section details the proposed framework for developing LSTM-based heartbeat classification models in distributed edge computing environments. We present four interconnected components: (1) the development of LSTM-based classifiers with preprocessing and feature extraction pipelines, (2) the design of classifier model architectures, (3) the training methodology and comprehensive performance metrics, and (4) the deployment strategy on NVIDIA Jetson Orin devices. Together, these components establish a comprehensive experimental pipeline, spanning from raw ECG data to deployed edge inference, which enables a thorough evaluation of both classification accuracy and resource efficiency in distributed systems. The following subsections provide detailed technical specifications for each component.

### 3.1. Making an LSTM-Based Heartbeat Classifier

This study develops an LSTM-based heartbeat classifier through a comprehensive machine learning workflow that encompasses data preprocessing, model training, and performance evaluation. The trained classifier is subsequently deployed on a Jetson device to enable real-time inference capabilities. The system is designed to predict heartbeat classifications using data captured from Polar H10 sensors, with rigorous testing scenarios implemented to validate the model’s accuracy and reliability under real-world operating conditions.

#### Source of Heartbeat Data

The dataset used in this study was obtained from the MIT-BIH Arrhythmia Database. The ECG data in the MIT-BIH Arrhythmia Database contains annotations consisting of 17 classes. The dataset contains 48 patient recordings, each featuring a 30-min ECG. Four recordings (numbers 102, 104, 107, and 217) were excluded from the training data because they contained paced rhythms, resulting in 44 recordings used for analysis.

To standardize the classification approach and align with international cardiac monitoring guidelines, the original 17 MIT-BIH beat annotations were reorganized according to the Association for the Advancement of Medical Instrumentation (AAMI) standard [13,21]. This widely accepted standard consolidates heartbeat types into five clinically meaningful categories: Normal (N), Supraventricular Ectopic Beat (S), Ventricular Ectopic Beat (V), Fusion (F), and Unknown (Q).

The feature extraction process employed in this study utilizes three complementary approaches: RR interval (RRI) features that capture temporal characteristics, morphological features representing the shapes of heartbeat waveforms, and wavelet transform features that provide time–frequency domain information. The explanations of each feature extraction are presented in Section 3.2.

### 3.2. Classification Features

The main features used in this study are the RR interval, morphology, and wavelet.

#### 3.2.1. RRI

The MIT-BIH Arrhythmia Database, R-peak annotations are already provided, indicating the position of the ‘R’ peak in the ECG signal. Based on these R-peak positions, the distance between two consecutive ‘R’ peaks can be calculated to obtain the RR interval. The RR-Interval is calculated using Equation (1):

(1) RRi[i]=Rpeak[i+1]−Rpeak[i]

where RRi[i] is the *i*-th RR-Interval, and Rpeak[i] is the *i*-th R-peak position. The RR-Interval is then extracted into 9 features, namely:

$$RR0,\;RR_{-1},\;RR_{+1},\;\frac{RR0}{\mathrm{avgRR}},\;tRR0,\;\frac{RR_{-1}}{\mathrm{avgRR}},\;\frac{RR_{-1}}{RR0},\;\frac{RR_{+1}}{\mathrm{avgRR}},\;\frac{RR_{+1}}{RR0}$$

Table 1 presents a detailed description of each feature used in this study. The average RR-Interval (avgRR) and the standard deviation of the RR-Interval (stddevRR) are calculated using Equations (2) and (3). The reason for using 42 beats as our starting point is based on our experimental observations to obtain stable data. ECG signals acquired from the Polar H10 sensor exhibit significant motion artifacts and startup artifacts during the initial recording period.

(2) $$\mathrm{avgRR}=\frac{1}{42}\sum_{i=0}^{41}RRi\left[i\right]$$

(3) $$\mathrm{stddevRR}=\sqrt{\frac{1}{42}\sum_{i=0}^{41}{\left(RRi\left[i\right]-\mathrm{avgRR}\right)}^{2}}$$

#### 3.2.2. Morphology

In the process of extracting morphological features from the ECG (electrocardiogram) signal, the approach focuses on identifying and segmenting the signal around the R-peak, which is a prominent feature of the QRS complex. This segmentation captures the essential shape and structure of the heartbeat waveform, which is critical for accurate classification.

The first step involves correcting the R-peak position. Although initial R-peak indices are provided through annotations, slight inaccuracies may exist. To address this, for each detected R-peak index $r_{i}$ , the algorithm searches for the maximum amplitude within a small symmetric window surrounding the peak. This correction window is defined as $\left[r_{i}-W_{\mathrm{corr}},r_{i}+W_{\mathrm{corr}}\right]$ , where $W_{\mathrm{corr}}$ is a narrow window (e.g., 20 samples) used only for local adjustment, not for morphological analysis. The corrected R-peak position $r_{i}^{\prime }$ is determined as:

(4) $$r_{i}^{\prime }=\arg\max_{k\in [r_{i}-W_{\mathrm{corr}},r_{i}+W_{\mathrm{corr}}]}X_{k}$$

where $X_{k}$ represents the amplitude of the ECG signal at position *k*.

Once the R-peak position is corrected, the next step is morphological segmentation. The ECG signal is segmented by extracting a window of samples around the corrected R-peak to capture the morphology of the heartbeat, including the P-wave, QRS complex, and T-wave. This segmentation window is defined using separate parameters: a left margin $W_{\mathrm{left}}$ and a right margin $W_{\mathrm{right}}$ , which can be asymmetric to better reflect the physiological structure (e.g., the T-wave duration is generally longer than the P-wave). The segment corresponding to the *i*-th heartbeat is given by:

(5) $$B_{i}=\left[X_{r_{i}^{\prime }-W_{\mathrm{left}}},\ldots ,X_{r_{i}^{\prime }},\ldots ,X_{r_{i}^{\prime }+W_{\mathrm{right}}}\right]$$

where $B_{i}$ is the morphological feature vector used for further classification or analysis. Each segment $B_{i}$ is then assigned to an AAMI heartbeat class based on the original annotation symbol.

#### 3.2.3. Wavelet

Feature extraction from the MIT-BIH Arrhythmia dataset, using the Discrete Wavelet Transform (DWT) aims to capture essential characteristics of the signal in the frequency domain. Mathematically, DWT decomposes a signal x[n] into two primary components: approximation coefficients and detail coefficients. This process involves applying a low-pass filter h[n] and a high-pass filter g[n] , followed by down-sampling.

The approximation coefficients at level *j* are computed using the equation:

(6) $$A_{j}\left[k\right]=\sum_{n}x\left[n\right]\cdot h\left[2k-n\right],$$

This captures the low-frequency components of the signal. Conversely, the detail coefficients are calculated as:

(7) $$D_{j}\left[k\right]=\sum_{n}x\left[n\right]\cdot g\left[2k-n\right],$$

Representing the high-frequency components.

The reconstruction of the original signal can be performed using the inverse DWT (IDWT) as follows:

(8) $$x\left[n\right]=\sum_{k}\left(A_{j}\left[k\right]\cdot h\left[n-2k\right]+D_{j}\left[k\right]\cdot g\left[n-2k\right]\right),$$

where h[n] and g[n] are the reconstruction filters corresponding to the low-pass and high-pass filters, respectively.

The decomposition is performed iteratively up to a certain level *L*, with each level providing a finer frequency resolution. The multi-level decomposition relationship can be expressed as:

(9) $$x\left[n\right]=A_{L}+\sum_{j=1}^{L}D_{j},$$

where $A_{L}$ is the final approximation coefficient, and $D_{j}$ are the detail coefficients at each level. The final wavelet decomposition result of the signal can be mathematically represented as:

(10) $$\mathrm{DWT}\left(x\left[n\right]\right)=\left\{A_{L},D_{L},D_{L-1},\ldots ,D_{1}\right\},$$

where $A_{L}$ is the approximation coefficient at the highest level *L*, and $D_{j}$ for j=1,2,…,L are the detail coefficients at each corresponding level. For feature extraction, typically only the approximation coefficients from the highest level $A_{L}$ are retained, as they represent the dominant patterns of the ECG signal and are more resilient to noise. Thus, the result of the DWT offers a compact and informative feature representation for classification or further analysis.

Following the AAMI EC57 standards, we processed the MIT-BIH Arrhythmia Database using a three-feature extraction approach that yielded 97,806 beats for RR-Interval (RRI) features (89.3% retention) and 100,708 beats for both morphological and wavelet features (92.0% retention) out of the original 109,494 beats, as presented in Table 2. Following standard practices [13,22], records 102, 104, 107, and 217 were excluded due to a predominance of paced rhythms and technical artifacts.

For RRI feature extraction, we applied a 42-beat sliding window, as described in Section 3.2, where the average RR interval (avgRR) and standard deviation (stddevRR) are computed from the preceding 42 beats (Equations (2) and (3) to extract the nine temporal features listed in Table 1. This window size ensures statistical stability for heart rate variability and provides normalized measures such as RR0/avgRR and tRR0.

Morphological and wavelet features share a common preprocessing pipeline that operates on a 500 ms window centered at each corrected R-peak (90 samples before and after, sampled at 360 Hz). R-peak refinement was performed within a ±20-sample search window (Equation (4)). These segmented beats were subsequently processed using discrete wavelet transform (DWT) decomposition (Equations (6)–(10)) to extract wavelet-domain features. Because morphological and wavelet processing use the same beat segments, they result in identical beat counts (100,708).

The difference in retention, 89.3% for RRI and 92.0% for morphological/wavelet—reflects the distinct computational constraints of each method: RRI-based features depend on a rolling window of valid previous beats. In contrast, morphological and wavelet features are derived on a per-beat basis. Significantly, these retention levels fall well within the range of previously published studies. For instance, de Chazal et al. [13] reported 90,126 Normal, 7008 VEB, and 2779 SVEB beats after filtering. Llamedo and Martínez [22] reported 89,972 Normal, 7001 VEB, and 2775 SVEB beats. Our morphological/wavelet beat distribution 90,102 Normal, 7008 VEB, and 2781 SVEB closely matches both studies, reinforcing the reproducibility of our preprocessing. In contrast, Kiranyaz et al. [23] retained fewer beats (e.g., 74,805 Normal), likely due to stricter segmentation criteria. Acharya et al. [24] reported 90,592 Normal, 7235 VEB, and 2781 SVEB beats, which are also in line with our results. These comparisons confirm that our data filtering and feature extraction protocols are consistent with the established literature and sufficiently rigorous for robust heartbeat classification.

### 3.3. LSTM Model Architecture

This subsection examines three LSTM-based architectures designed explicitly for ECG heartbeat classification: LSTM-Sequential, Bi-LSTM, and LSTM-FCN. The models were created for the classification of Normal (N), Supraventricular Ectopic Beat (S), Ventricular Ectopic Beat (V), Fusion (F), and Unknown (Q), as shown in Table 2. Each architecture employs different strategies to capture temporal patterns in ECG signals, ranging from unidirectional sequential processing to bidirectional analysis and hybrid approaches that combine recurrent and fully connected layers.

#### 3.3.1. LSTM

We utilize the TensorFlow API through its high-level Keras interface to build and train an LSTM-based neural network model. TensorFlow is a widely used open-source framework for machine learning and deep learning, offering robust tools for model development and deployment. In this scenario, the *Sequential* API from TensorFlow’s Keras module is used, which allows for the straightforward construction of models by linearly stacking layers.

As shown in Table 3, the model begins with an *LSTM* layer, designed to handle sequential data by capturing long-term dependencies through its specialized memory cells. This is particularly useful for time-series data or sequences where past information is relevant to future predictions. The input shape *(9,1)* specifies that the model will process sequences with 9 time steps, each containing a single feature. The *Dense* layers that follow are fully connected layers that help refine the learned features from the LSTM layer. The use of the *tanh* activation function in the first *Dense* layer enables non-linear transformations, thereby enhancing the model’s ability to capture complex patterns. The final *Dense* layer with a *softmax* activation function is configured for multi-class classification, enabling the model to output probabilities for each of the five classes. The model is compiled with the *adam* optimizer, which is effective for most deep learning models due to its adaptive learning rate capabilities. The loss function, *categorical_crossentropy*, is chosen because it is suitable for scenarios where the target variable is one-hot encoded for multi-class classification.

#### 3.3.2. BI-LSTM

The BI-LSTM is constructed using the *Sequential* API, which allows for the straightforward stacking of layers as shown in Table 4. The first layer is a *Bidirectional* wrapper around an *LSTM* layer with 256 units. The bidirectional configuration enables the model to process input data in both forward and backward directions, enhancing its ability to capture contextual information from the entire sequence. Following the LSTM layer, a *Dense* layer with 256 units and a *tanh* activation function is included. This layer facilitates learning complex, non-linear relationships from the processed sequential data. The final *Dense* layer employs a *softmax* activation function, which is well-suited for multi-class classification problems, allowing the model to output probabilities for five distinct classes.

The model is compiled using the *adam* optimizer, known for its adaptive learning rate, making it efficient for training deep learning models. The loss function, *categorical_crossentropy*, is appropriate for multi-class classification scenarios where the labels are one-hot encoded.

#### 3.3.3. LSTM-FCN

We utilized a hybrid neural network model that combines Long Short-Term Memory (LSTM) and Fully Convolutional Network (FCN) architectures, commonly referred to as an LSTM-FCN model, as shown in Table 5. The model begins with an *Input* layer, accepting input sequences of shape *(MAX_SEQUENCE_LENGTH, 1)*, where each sequence has a specified length and a single feature per time step. The LSTM branch processes the input through an *LSTM* layer with *NUM_CELLS* units, designed to capture long-term temporal dependencies in the sequential data. A *Dropout* layer with a high dropout rate of 0.8 is applied to prevent overfitting by randomly deactivating a significant portion of neurons during training.

Simultaneously, the input is processed through a convolutional branch. The input tensor is first transformed using the *Permute* layer to adjust its dimensions, enabling compatibility with convolutional operations. The first *Conv1D* layer applies 128 filters with a kernel size of 8, using ’same’ padding to maintain the input shape. The *he_uniform* kernel initializer is employed to optimize weight initialization for better convergence. A *BatchNormalization* layer follows to stabilize and accelerate training, succeeded by a *ReLU* activation function for introducing non-linearity. The process is repeated with a second *Conv1D* layer using 256 filters and a kernel size of 5, followed by batch normalization and ReLU activation. A third *Conv1D* layer with 128 filters and a kernel size of 3 continues this process, further refining feature extraction.

After the convolutional operations, a *GlobalAveragePooling1D* layer is applied to reduce the spatial dimensions and aggregate features, ensuring the model is invariant to the temporal length of the input. The outputs from the LSTM and convolutional branches are concatenated using the *concatenate* layer, combining temporal and spatial features for a richer representation. The final layer is a *Dense* layer with *NB_CLASS* units and a *softmax* activation function, suitable for multi-class classification as it outputs a probability distribution over the classes.

The model is instantiated using TensorFlow’s *Model* class, linking the input layer to the final output layer. This hybrid approach leverages the sequential learning capabilities of LSTM and the hierarchical feature extraction strength of FCN, making it particularly effective for sequence classification tasks that require both temporal and spatial feature understanding.

### 3.4. Training Process and Performance Metrics

All models were trained using the TensorFlow framework. The training was conducted using a training dataset comprising 70% of the total data. The training used 50 epochs to provide sufficient time for the model to learn patterns without overfitting, while allowing monitoring of progress and early stopping if needed. A batch size of 256 was chosen to efficiently utilize GPU memory and provide stable gradient updates while balancing computational speed and model performance.

Hyperparameters were selected through a preliminary grid search. A batch size of 256 was chosen from 64, 128, 256, 512 as it optimized GPU memory utilization while maintaining gradient stability. Training was limited to 50 epochs with early stopping based on observed convergence patterns around epochs 35–45. The Adam optimizer (learning rate = 0.001) was selected after comparing rates of 0.01, 0.001, 0.0001, with categorical cross-entropy as the loss function and dropout (0.2) for regularization. The evaluation of LSTM, Bi-LSTM, and LSTM-FCN models was conducted using a test dataset consisting of 30% of the total dataset. Several evaluation metrics were used in this study, including accuracy, precision, recall, and F1-score.

#### 3.4.1. Training Model Using RRI

Table 6 presents the evaluation results of three deep learning models—LSTM-Sequential, Bi-LSTM, and LSTM-FCN—using the RRI feature. Among the three models, LSTM-Sequential achieves the highest accuracy (0.965), indicating superior overall classification performance. This model also demonstrates the highest precision (0.963) and recall (0.965), indicating that it excels at minimizing both false positives and false negatives. The F1-score of 0.962 confirms the balanced performance between precision and recall.

Bi-LSTM shows slightly lower but competitive results with an accuracy of 0.963, precision of 0.962, and recall of 0.963. Its F1-score of 0.962 matches that of LSTM-Sequential, indicating similarly balanced performance, albeit with marginally lower individual metrics.

LSTM-FCN achieves the same accuracy (0.963) and recall (0.963) as Bi-LSTM but exhibits slightly lower precision (0.960), resulting in a lower F1-score (0.960). This suggests that while LSTM-FCN correctly identifies positive cases at a similar rate to Bi-LSTM, it produces slightly more false positives.

Table 7 presents the class-wise accuracy results of three deep learning models (LSTM-Sequential, Bi-LSTM, and LSTM-FCN) using RRI features for classifying five types of cardiac arrhythmias (N, S, V, F, Q). All models demonstrate excellent performance on Normal beats (N) with an accuracy of 0.98-0.99, but struggle with Fusion (F) and Unknown (Q) classes, which show low accuracy due to limited sample data.

#### 3.4.2. Training Model Using Morphology

Table 8 presents the evaluation results of three deep learning models—LSTM-Sequential, Bi-LSTM, and LSTM-FCN—using the Morphology feature.

Among the three models, Bi-LSTM achieves the highest accuracy (0.988) and recall (0.988), demonstrating superior ability in correctly identifying positive instances. However, its precision (0.987) matches that of LSTM-Sequential, resulting in both models sharing the same F1-score (0.987). This indicates that while Bi-LSTM excels at minimizing false negatives, both models maintain a similar balance between precision and recall.

LSTM-Sequential demonstrates remarkably consistent performance with all metrics at 0.987 (accuracy, precision, recall, and F1-score). This uniformity across metrics suggests highly balanced and stable classification performance, making it exceptionally reliable for applications where consistent behavior is crucial.

LSTM-FCN shows slightly lower but uniform performance across all metrics (0.985), indicating consistent but marginally less effective classification compared to the other two models. The identical values across all metrics suggest balanced performance without bias toward either precision or recall.

Table 9 presents the class-wise accuracy results of three deep learning models (LSTM-Sequential, Bi-LSTM, and LSTM-FCN) using morphology features for classifying five types of cardiac arrhythmias (N, S, V, F, Q). Compared to RRI features, morphology features significantly improve performance across most classes, particularly for Fusion beats (F), where accuracy reaches 0.70-0.84, and some models can detect Unknown beats (Q) with 0.20 accuracy.

#### 3.4.3. Training Model Using Wavelet

Table 10 presents the evaluation results of three deep learning models—LSTM-Sequential, Bi-LSTM, and LSTM-FCN—using the Wavelet feature. Among the three models, LSTM-Sequential demonstrates exceptional and perfectly balanced performance across all metrics, with values of 0.990 (accuracy, precision, recall, and F1-score). This uniformity indicates that the model excels equally at minimizing both false positives and false negatives while maintaining the highest overall classification accuracy.

LSTM-FCN achieves the same high accuracy (0.990) and recall (0.990) as LSTM-Sequential, indicating equivalent ability in correctly identifying positive instances. However, its precision drops to 0.980, resulting in a lower F1-score (0.980). This discrepancy suggests that while LSTM-FCN correctly identifies positive cases at the same rate as LSTM-Sequential, it generates more false positives, resulting in reduced precision.

Bi-LSTM shows consistent but slightly lower performance across all metrics (0.987), positioning it as the least effective model among the three. However, the balanced nature of its metrics indicates stable classification performance without bias toward either precision or recall.

Table 11 presents the class-wise accuracy results of three deep learning models (LSTM-Sequential, Bi-LSTM, and LSTM-FCN) using Wavelet features for classifying five types of cardiac arrhythmias (N, S, V, F, Q). Wavelet features demonstrate consistent performance across all models, with excellent accuracy for Normal (N) and Ventricular (V) beats, moderate performance for Supraventricular (S) and Fusion (F) beats, and improved detection of Unknown (Q) beats, achieving 0.20 accuracy compared to RRI features.

The evaluation results of the three deep learning models—LSTM-Sequential, Bi-LSTM, and LSTM-FCN—across different feature sets (RRI, Morphology, and Wavelet) provide comprehensive insights into their classification performance through multiple evaluation metrics.

When using the RRI feature, LSTM-Sequential achieves the highest accuracy (0.965) and precision (0.963), with superior recall (0.965) compared to the other models. While all three models share similar F1-Scores (0.962 for LSTM-Sequential and Bi-LSTM, 0.960 for LSTM-FCN), LSTM-Sequential’s marginally better individual metrics make it the most effective choice for RRI-based classification.

For the Morphology feature, all models demonstrate exceptional performance with minimal variations. Bi-LSTM leads with the highest accuracy (0.988) and recall (0.988), while maintaining precision (0.987) equal to LSTM-Sequential. Notably, LSTM-Sequential exhibits perfectly balanced metrics (all at 0.987), and LSTM-FCN shows consistent but slightly lower performance across all metrics (0.985). The F1-Scores reflect this pattern, with Bi-LSTM and LSTM-Sequential both achieving 0.987, while LSTM-FCN scores 0.985.

The Wavelet feature reveals interesting performance patterns. LSTM-Sequential achieves a perfect balance, with all metrics at 0.990, representing optimal performance. While LSTM-FCN matches LSTM-Sequential in accuracy (0.990) and recall (0.990), its significantly lower precision (0.980) results in a reduced F1-score (0.980), indicating a higher rate of false positives. Bi-LSTM maintains consistent performance across all metrics at 0.987.

Overall performance analysis reveals that LSTM-Sequential emerges as the most robust model, achieving the best or near-best results across all feature sets. It particularly excels with RRI and Wavelet features, where it demonstrates superior balanced performance. Bi-LSTM shows its strength with Morphology features, achieving the highest accuracy and recall, making it ideal when minimizing false negatives is crucial. LSTM-FCN, while competitive in accuracy, exhibits weaknesses in precision, particularly with Wavelet features, suggesting it may be less suitable for applications where minimizing false positives is crucial.

The minimal performance differences across models (typically within 0.5%) suggest that all three architectures are highly effective for ECG signal classification. The choice of model should consider not only accuracy but also the balance between precision and recall based on specific application requirements, computational constraints, and the relative costs of false positives versus false negatives.

### 3.5. Deployment on Jetson

In the inference process, several stages are carried out, including preprocessing, R-peak detection, feature extraction, and prediction. Preprocessing is performed to clean the data from noise and prepare it for the following stages. The preprocessing process consists of resampling, removing high-frequency noise using wavelet denoising, removing baseline wander, and normalizing the data using z-score normalization. The ECG data used for testing is recorded using the Polar H10 device. The ECG data recorded with the Polar H10 has a sampling frequency of 130 Hz, whereas the dataset used to train the model has a sampling frequency of 360 Hz. Therefore, the recorded ECG data needs to be converted to 360 Hz to be compatible with the trained model. The ECG recording data is resampled to 360 Hz using the resampling method. This resampling process is performed using the resample function provided by the SciPy library.

**Figure 2:**
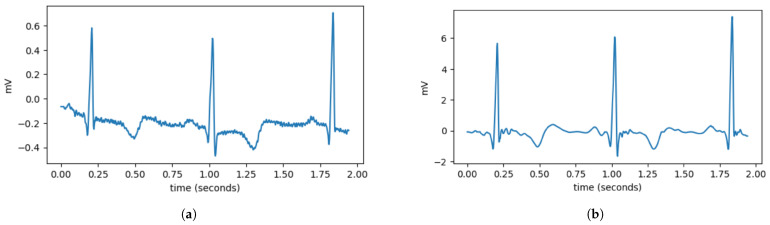
Comparison of ECG data before and after preprocessing. (**a**) It can be observed that the ECG data still contain disturbances, such as high-frequency noise and variations in the signal baseline. (**b**) After preprocessing, the ECG signal becomes smoother and has a more stable baseline.

During the data acquisition process, ECG signals are highly susceptible to high-frequency noise. High-frequency components in ECG data are considered noise that can interfere with the heartbeat classification process. In this study, high-frequency noise is removed using the wavelet denoising method. As depicted in Figure 2, wavelet denoising removes noise from signals using the wavelet transform. Wavelet transform enables the signal to be decomposed into multiple frequency components, allowing for noise removal at specific frequencies. The wavelet denoising method employed in this study is VisuShrink with soft thresholding mode, utilizing the Daubechies 8 (db8) wavelet with 10 levels of wavelet decomposition. Apart from high-frequency noise, ECG signals are also susceptible to baseline wander (BW). Baseline wander is a low-frequency noise present in ECG signals, which can be caused by factors such as respiration, electrically charged electrodes, and patient movement. Baseline wander is removed by subtracting the ECG signal from its trend signal. The trend signal is obtained using a double median filter with window sizes of 71 and 215.

The double median filter theory utilizes the unique properties of median filtering at different scales to separate the baseline wander from the ECG signal effectively. The first median filter, with a window size of 71 samples, serves to remove impulse noise while preserving the sharp edges characteristic of ECG waveforms, particularly the QRS complexes. This initial filtering stage eliminates high-frequency artifacts while maintaining the morphological integrity of the cardiac signal. The second median filter with a larger window size of 215 samples is designed to extract the low-frequency baseline trend. This wider window captures the slow variations associated with respiratory movement and electrode drift without being influenced by the faster cardiac components.

The median filter (11) replaces each data point in the signal with the median value within the specified window. By applying these filters sequentially, the method effectively eliminates baseline drift while preserving essential ECG features. The combination of these two window sizes (71 and 215 samples) is optimized for typical ECG sampling rates and provides robust baseline correction across various physiological conditions and noise environments.

(11) $$y\left[n\right]=\mathrm{median}\left(x\left[n-\frac{M}{2}:n+\frac{M}{2}\right]\right)$$

where y[n] is the signal after applying the median filter, x[n] is the original signal, and *M* is the window length of the median filter. After removing noise and baseline wander, the next step is to normalize the data. Normalization is performed to avoid differences in data scale. The normalization method used in this study is Z-score normalization. Z-score normalization transforms the data into a normal distribution with a mean of 0 and a standard deviation of 1. Z-score normalization is defined by the following Equation (12). Where *z* is the normalized data, *x* is the original data, μ is the mean of the data, and σ is the standard deviation of the data.

(12) $$z=\frac{x-\mu }{\sigma }$$

After the preprocessing process is completed, R-peak detection is performed to determine the position of R-peaks in the ECG signal. R-peak detection is carried out using the Pan–Tompkins algorithm. The Pan–Tompkins algorithm is a method used to detect R-peaks in ECG signals [25]. From the detected R-peak positions, the distance between R-peaks is calculated to obtain classification features, including RR-interval, Morphology, and Wavelet. Next, the inference is adjusted according to the model obtained from the previous training. In our experimental study, we ran the inference for all models.

Regarding the deployment of the classifier model into our system, the trained TensorFlow models initially exist in H5 format, which is unoptimized and results in large file sizes that are inefficient for deployment on resource-constrained devices. To address this limitation, the models need to be converted to TensorFlow Lite (TFLite) format, which is designed explicitly for inference on devices with limited computational resources. The conversion process utilizes TFLiteConverter, a TensorFlow API that transforms standard TensorFlow models into the optimized TFLite format. During conversion, dynamic range quantization is applied as an optimization technique, reducing the precision of numbers representing model parameters to decrease file size and improve inference performance without requiring a representative dataset for calibration. When converting LSTM models to TFLite format, specific challenges arise due to their dynamic batch size requirements and dependency on built-in TensorFlow operations. This creates an unwanted dependency on the TensorFlow runtime, which contradicts the goal of creating a lightweight, standalone model. The solution involves setting the batch size to 1, enabling the TFLite model to run independently without TensorFlow dependencies. The conversion results demonstrate remarkable efficiency gains, with model sizes reduced by approximately 90% across all architectures. These substantial size reductions translate to reduced storage requirements, faster model loading times, and more efficient memory usage during inference operations on the edge device.

**Figure 3:**
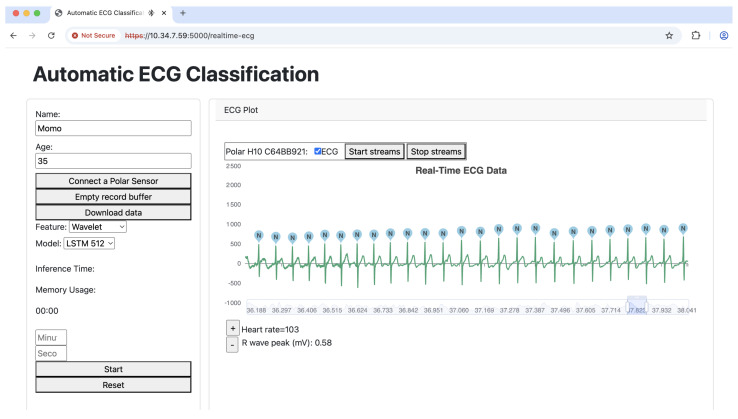
Our inference system.

Figure 3 illustrates the real-time inference process of the automatic ECG classification system running on the Jetson Orin. The system continuously processes ECG data streamed from the Polar H10 heart rate sensor, performing real-time classification of each detected heartbeat. The Flask-based backend facilitates communication between the front end and the deep learning model deployed on the Jetson Orin, utilizing the LSTM 512 model for classification. The wavelet transform is employed as the feature extraction technique, enabling the model to capture essential characteristics of the ECG signal for accurate classification. The results are then visualized in a dynamic chart, where each detected heartbeat is labeled according to its predicted class. This approach ensures an efficient and responsive inference pipeline, making the system suitable for real-time ECG monitoring.

## 4. Performance of Real-Time Heartbeat Classification on Jetson

This section evaluates the real-time performance of LSTM-based heartbeat classification models deployed on NVIDIA Jetson Orin edge devices. We present a comprehensive resource utilization analysis covering hardware specifications, CPU and GPU usage patterns, memory consumption, and inference time measurements. These metrics are essential for understanding the practical deployment constraints and optimization opportunities in distributed edge computing environments. The evaluation reveals critical trade-offs between model complexity, classification accuracy, and computational efficiency.

### 4.1. Hardware Spesication

The NVIDIA Jetson Orin Developer Kit is a powerful AI edge computing platform designed for robotics, autonomous systems, and AI workloads. As shown in Table 12, it offers high computational power with energy efficiency, making it suitable for deep learning and real-time inference tasks. The platform is particularly well-suited for LSTM-based models due to its optimized CUDA libraries, which accelerate recurrent computations essential for processing the sequential nature of ECG signals. The Jetson’s Deep Learning Accelerator (DLA) and GPU work synergistically to handle the temporal dependencies in LSTM networks. At the same time, its 32 GB unified memory accommodates the storage requirements of multiple LSTM hidden states during inference. Furthermore, the platform’s TensorRT optimization engine offers specialized optimizations for LSTM layers, including kernel fusion and precision calibration, which significantly reduce inference latency while maintaining classification accuracy—essential requirements for real-time heartbeat monitoring in clinical edge deployments.

### 4.2. CPU Usage

The experiment evaluated the CPU usage of ECG classification models running on Jetson Orin, using ECG data from the Polar H10 sensor. Several classifier models were tested across different feature representations, namely RRI, Morphological, and Wavelet features, with varying configurations (512, 256, and FCN).

**Figure 4:**
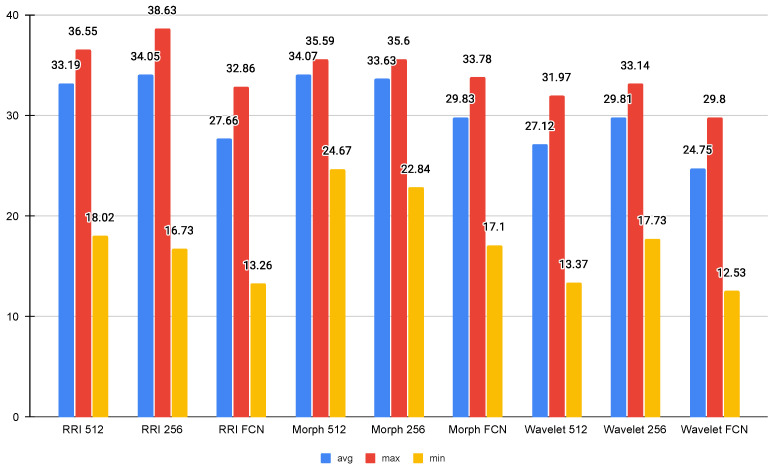
Average CPU usage (%).

As depicted in Figure 4, the Wavelet FCN model demonstrated the lowest CPU usage, with an average of 24.75%, a maximum of 29.80%, and a minimum of 12.53%, indicating its efficiency in resource utilization. On the other hand, the RRI 256 model had the highest average CPU usage at 34.05%, while the RRI 512 and Morph 512 models also exhibited relatively high CPU usage at 33.19% and 34.07%, respectively. The maximum CPU usage was recorded at 38.63% by the RRI 256 model, suggesting it places a higher computational demand on the Jetson Orin.

Comparing FCN-based models with their non-FCN counterparts, it is evident that FCN models generally consume less CPU, as seen in all three feature types (RRI, Morph, and Wavelet). This implies that FCN-based architectures are more optimized for efficient inference. Additionally, the minimum CPU usage values suggest that the system experiences significant fluctuations in load, with FCN-based models consistently requiring the least amount of processing power at their lowest points. The results indicate that Wavelet-based FCN models offer the most efficient CPU usage.

### 4.3. GPU Usage

**Figure 5:**
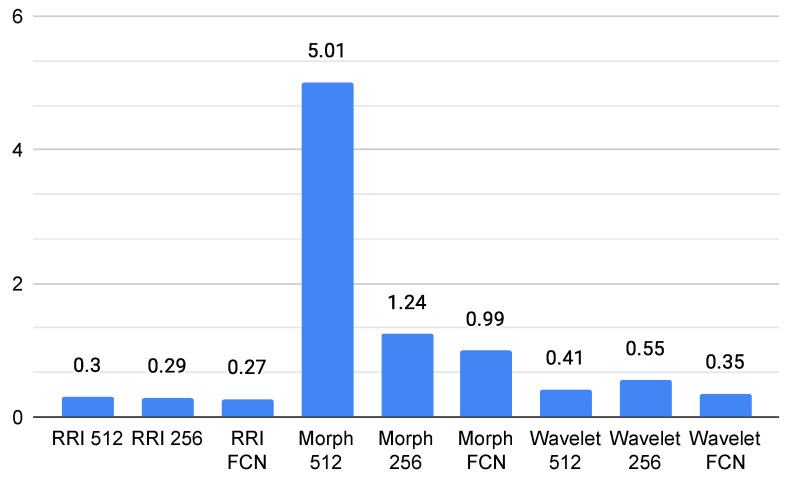
Average GPU usage (%).

The GPU usage analysis for ECG classification models on the Jetson Orin reveals significant variations across different feature representations and model architectures, as shown in Figure 5. The Morph 512 model exhibits the highest GPU consumption, with an average usage of 5.01% and a peak of 98.9%, indicating that morphological features, especially at higher resolutions, require substantial computational power. In contrast, RRI-based and Wavelet-based models maintain significantly lower GPU usage, with averages remaining below 1%. Among them, Wavelet FCN appears the most efficient, averaging 0.34% GPU usage. The Fully Convolutional Network (FCN) models consistently demonstrate lower GPU usage across all feature types, suggesting better optimization for edge computing applications.

**Figure 6:**
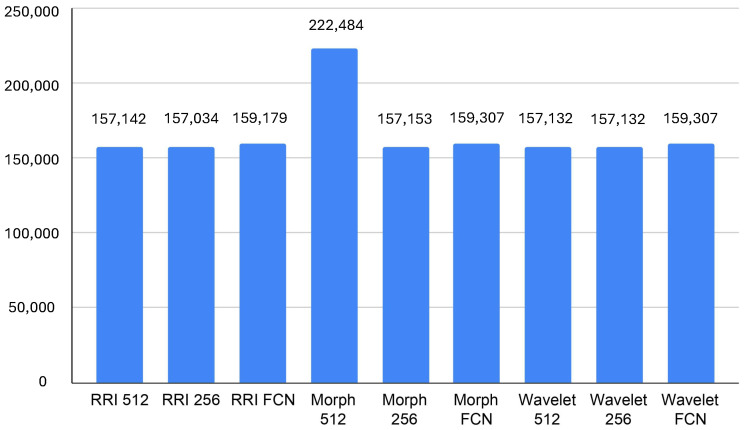
Average GPU memory usage (KB).

In terms of GPU memory, according to Figure 6, the Morph 512 model also stands out, consuming an average of 222,484 KB, which is notably higher than other models. The RRI-based and Wavelet-based models remain within a relatively stable range of 157,132 KB to 159,308 KB, indicating moderate memory consumption. FCN-based models, while slightly increasing memory usage, contribute to reduced GPU processing demands, making them a viable choice for real-time inference. The findings suggest that, for resource-constrained edge environments, Wavelet FCN or RRI FCN may offer the best trade-off between computational efficiency and classification performance. In contrast, Morph 512, despite its potential accuracy benefits, could be too resource-intensive for real-time deployment.

### 4.4. Memory Usage

**Figure 7:**
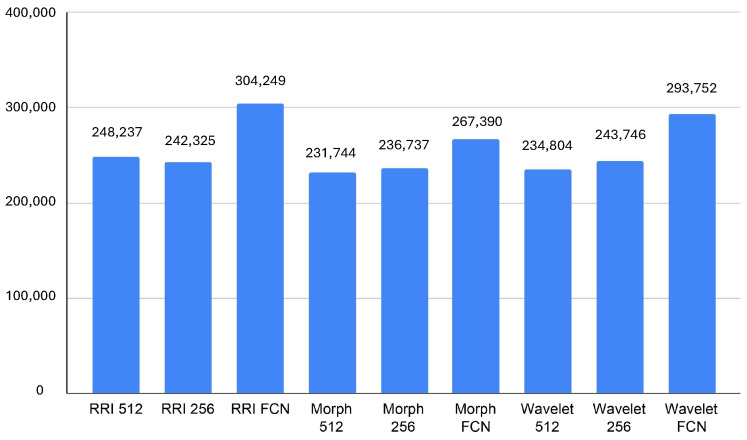
Average memory usage (KB).

The memory usage analysis across different models highlights notable differences in resource consumption. It can seen in Figure 7, The RRI FCN and Wavelet FCN models exhibit the highest average memory usage, reaching 304,249 KB and 293,752 KB, respectively, with peak values exceeding 310,000 KB. This suggests that these architectures, while possibly delivering superior feature extraction and classification capabilities, demand significant memory, which could impact real-time performance on edge devices like the Jetson Orin. In contrast, Morph 512 and Morph 256 consume the least memory, averaging 231,744 KB and 236,737 KB, respectively, indicating better efficiency in memory management despite their relatively higher GPU utilization.

The minimum memory usage values further reinforce these trends, with RRI FCN and Wavelet FCN requiring at least 266,807 KB and 278,088 KB, respectively, while Morph 512 and Morph 256 operate with as little as 212,083 KB and 217,752 KB. These insights suggest that while FCN-based models might be more effective in classification, they trade off efficiency with high memory requirements. Therefore, for real-time applications on resource-limited devices, Morph-based models might be more suitable due to their lower memory footprint, provided that their accuracy is sufficient for the target task.

### 4.5. Inference Time

**Figure 8:**
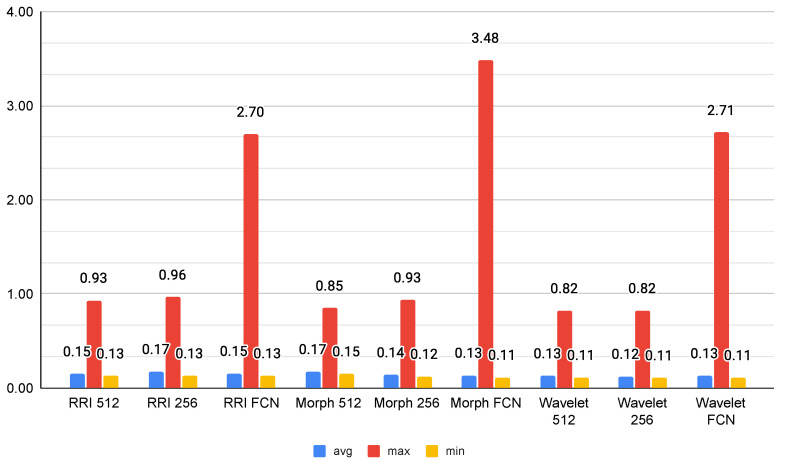
Average inference time (s).

As shown in Figure 8, the inference time results suggest that Wavelet-based models exhibit the most efficient performance, with Wavelet 256 achieving the lowest average inference time at 0.1224 s, closely followed by Wavelet 512 (0.1280 s). This efficiency makes wavelet transformations a strong candidate for real-time applications, particularly in scenarios where rapid decision-making is required. Conversely, FCN-based models demonstrate the highest variability, with RRI FCN and Wavelet FCN reaching maximum inference times of 2.69 s and 2.71 s, respectively. These peaks suggest that fully connected layers introduce significant computational overhead, making them less ideal for low-latency applications.

Among the morphological models, Morph 256 emerges as an optimal balance between speed and complexity, with an average inference time of 0.1384 s. However, Morph FCN shows an extreme maximum inference time of 3.48 s, indicating occasional heavy processing loads. RRI-based models maintain moderate inference times; however, their FCN variant exhibits increased processing time, likely due to the additional feature transformations. Overall, Wavelet 256 stands out as the best candidate for efficient real-time inference, while FCN models, despite their potential accuracy benefits, may be unsuitable for applications with strict latency constraints.

## 5. Discussion

Our study implements multi-class classification for five ECG arrhythmia classes, strictly adhering to the AAMI standard: Normal (N), Supraventricular ectopic beats (S), Ventricular ectopic beats (V), Fusion beats (F), and Unknown beats (Q). To comprehensively evaluate the classification performance, we tested three deep learning models—LSTM-Sequential, Bi-LSTM, and LSTM-FCN—across different feature sets (RRI, Morphology, and Wavelet).

The evaluation results reveal distinct performance patterns across these feature–model combinations. When classifying the five AAMI-standard arrhythmia classes using the RRI feature, LSTM-Sequential achieves the highest accuracy (96.5%) and precision (96.3%), with superior recall (96.5%) compared to the other models. While all three models share similar F1-scores (96.2% for LSTM-Sequential and Bi-LSTM, 96% for LSTM-FCN), LSTM-Sequential’s marginally better individual metrics make it the most effective choice for RRI-based arrhythmia classification.

For the Morphology feature in distinguishing between N, S, V, F, and Q classes, all models demonstrate exceptional performance with minimal variations. Bi-LSTM leads with the highest accuracy (98.8%) and recall (98.8%), while maintaining precision (98.7%) equal to LSTM-Sequential. Notably, LSTM-Sequential exhibits perfectly balanced metrics (all at 98.7%), and LSTM-FCN shows consistent but slightly lower performance across all metrics (98.5%). The F1-Scores reflect this pattern, with Bi-LSTM and LSTM-Sequential both achieving 0.987, while LSTM-FCN scores 98.5%.

The Wavelet feature reveals interesting performance patterns in classifying the five arrhythmia types. LSTM-Sequential achieves a perfect balance, with all metrics at 99%, representing optimal performance for multi-class arrhythmia classification. While LSTM-FCN matches LSTM-Sequential in accuracy (99%) and recall (99%), its significantly lower precision (98%) results in a reduced F1-Score (98%), indicating a higher rate of false positives when distinguishing between the AAMI-standard classes. Bi-LSTM maintains consistent performance across all metrics, achieving a score of 98.7%.

Overall performance analysis for the five-class ECG arrhythmia classification reveals that LSTM-Sequential emerges as the most robust model, achieving the best or near-best results across all feature sets. It particularly excels with RRI and Wavelet features, where it demonstrates superior balanced performance in distinguishing Normal, Supraventricular ectopic, Ventricular ectopic, Fusion, and Unknown beats. Bi-LSTM shows its strength with Morphology features, achieving the highest accuracy and recall, making it ideal when minimizing false negatives in arrhythmia detection is crucial. LSTM-FCN, while competitive in accuracy, exhibits weaknesses in precision, particularly with Wavelet features, suggesting it may be less suitable for clinical applications where minimizing false positives in multi-class arrhythmia classification is crucial.

The deployment evaluation of the Jetson Orin highlights significant trade-offs between accuracy and computational efficiency within both single-node and distributed system contexts. Despite the superior accuracy of Wavelet-based models, their computational efficiency becomes a critical consideration for edge computing platforms. The inference time results show that Wavelet 256 has the lowest average inference time (0.1224 s), making it highly suitable for real-time applications. Conversely, FCN-based architectures exhibited significantly higher maximum inference times (up to 2.7 s), suggesting that while fully connected networks may enhance feature extraction, they introduce substantial computational overhead.

Real-time heartbeat classification is essential for detecting sudden arrhythmias that require immediate response. Prior studies showed that processing 600 s of ECG data could take up to 14.8 s, while even shorter inputs like 60 and 10 s required 2.8 and 0.9 s, respectively. Although such delays may be tolerable for offline analysis, they are not suitable for time-critical scenarios where early warning can determine patient outcomes.

In our evaluation, the classifier using wavelet-based features with 256 decomposition levels achieved the lowest average inference time—only 0.1224 s. This performance is well within the range of human heartbeat intervals, which typically range from 0.6 to 1 s. As such, this model is capable of producing predictions faster than the time between two heartbeats, making it highly suitable for real-time ECG inference using stream processing.

The comprehensive resource utilization analysis reveals distinct patterns across different model architectures. CPU usage analysis demonstrates that Wavelet FCN models achieve the most efficient resource utilization, with an average CPU usage of 24.75%, making them particularly suitable for distributed deployment scenarios. In contrast, RRI-based models exhibited the highest CPU consumption at 34.05% average usage, which could limit their scalability in distributed architectures. GPU utilization patterns reveal even more pronounced differences, with Morph 512 models exhibiting the highest GPU consumption at 5.01% average usage and peaks reaching 98.9%. Conversely, Wavelet FCN models exhibit minimal GPU usage, averaging 0.34%, making them highly suitable for distributed architectures where multiple models can operate simultaneously on shared hardware resources.

Memory consumption analysis further emphasizes the importance of selecting resource-efficient models in distributed systems. RRI FCN and Wavelet FCN models require substantial memory resources, averaging 304,249 KB and 293,752 KB, respectively, while Morph-based models demonstrate superior memory efficiency. These resource utilization characteristics have significant implications for distributed system design, where efficient resource usage enables horizontal scaling and effective resource sharing across multiple inference processes.

From a practical deployment perspective, the Wavelet-Bi-LSTM model emerges as the best candidate for distributed edge computing on Jetson Orin platforms, offering a strong compromise between classification accuracy, resource efficiency, and scalability. The minimal performance differences across models (typically within 0.5%) suggest that all three architectures are highly effective for ECG signal classification. However, the choice of model should consider not only accuracy but also the balance between precision and recall based on specific application requirements, computational constraints, and the relative costs of false positives versus false negatives.

The web service architecture implemented in this study provides additional flexibility for distributed deployment through RESTful API design, enabling easy horizontal scaling and dynamic resource allocation. Future optimizations could focus on heterogeneous model distribution, where different feature extraction methods and model architectures are deployed across nodes based on their specific resource characteristics and performance requirements. These findings highlight the importance of striking a balance between accuracy and resource efficiency when designing distributed real-time ECG classification systems, which must operate concurrently within limited hardware resources.

### Comparison with Previous Works

We primarily focused on evaluating LSTM-based models (LSTM-Sequential, Bi-LSTM, and LSTM-FCN) for ECG signal classification on Jetson Orin platforms, achieving a leading 99% accuracy with Wavelet-based features. This performance is comparable to or surpasses other advanced models, such as Sun et al.’s CNN-LSTM-SE (98.5% accuracy) [17], Farag’s Tiny Matched Filter-Based CNN (98.18% average accuracy) [20], and Liu et al.’s Lightweight 1D-CNN (99.59% accuracy in software) [14], while significantly outperforming simpler methods like KNN (81–97% class-specific accuracy) [15]. For real-time performance, our Wavelet 256 models demonstrated an average inference time of 0.1224 s (122.4 ms) on the Jetson Orin, which is competitive with or faster than many deep learning deployments on edge GPUs. While ultra-optimised models for highly constrained microcontrollers achieve sub-millisecond inference (e.g., Farag’s < 1 ms on Raspberry Pi [20], Hizem et al.’s 0.063 ms on host/0.200 ms on Raspberry Pi 4 [16]) or An et al.’s 19 ms on STM32F429 [18], and hardware-optimised FPGA solutions like Liu et al.’s LW-CNN achieve 63 ms [14] or another work (Tang et al.) reports CNN-LSTM around 208.2 ms, as noted in [14], our work targets efficient use of more powerful edge GPUs. Our resource utilisation analysis highlighted Wavelet FCN models’ CPU efficiency (average 24.75%) and minimal GPU usage (average 0.34%), crucial for distributed architectures, contrasting with the extreme model compression (e.g., Farag’s 15 KB model size [20], An et al.’s 1242.58 times smaller model for microcontrollers [18], Hizem et al.’s 193.359 KB optimised CNN [16], and Liu et al.’s 11,736 parameter, multiplication-free FPGA design [14]) seen in studies focused on less capable hardware. Our study’s unique contributions include its rigorous analysis of feature selection impacts and a specific focus on distributed edge computing, horizontal scaling, dynamic resource allocation, and heterogeneous model distribution via RESTful API design, distinguishing it from other papers which focus on ECG image detection (Ma & Zhang’s Mamba-RAYOLO) [19], or specific TinyML optimizations for microcontrollers (Hizem et al.’s TinyML [16], An et al.’s knowledge distillation for single-lead ECG [18]).

## 6. Conclusions and Future Work

This study presents the design and evaluation of a real-time heartbeat classification system deployed on distributed edge devices using Long Short-Term Memory (LSTM)-based models. By leveraging stream processing and a distributed RESTful architecture, the system addresses the latency and scalability limitations of traditional batch-based ECG classification methods. Through the integration of Polar H10 sensors, the Web Bluetooth API, and a Flask-based backend, the platform enables continuous ECG monitoring with real-time feedback and low-latency inference, making it suitable for on-device deployment.

A comprehensive evaluation across multiple features and model architectures reveals that wavelet-based features consistently provide the best balance between classification performance and computational efficiency. In particular, the LSTM-Sequential model with wavelet features achieves near-perfect classification results (accuracy, precision, recall, and F1-score all above 99%) with an average inference time of only 0.1224 s. This is significantly faster than the average human heartbeat interval (0.6–1.0 s), confirming the model’s ability to operate within real-time constraints and detect five types of heartbeats before the next heartbeat occurs. In contrast, FCN-based models—though accurate—require inference times exceeding 2 s, limiting their practicality for time-sensitive applications.

Resource utilization analysis on Jetson Orin devices confirms the feasibility of deploying lightweight models in distributed environments. The RESTful API design supports dynamic load balancing across nodes, enhancing system resilience. The findings validate that real-time inference is not only technically achievable but also clinically necessary, as it enables the immediate detection of life-threatening cardiac events—something post-analysis cannot offer. Future work will extend this study by developing a real-world dataset collected directly from Polar H10 devices, including data from arrhythmic patients, to further validate the system under more diverse and realistic conditions.

## Figures and Tables

**Table 1:** Description of RR interval.

Feature	Description
RR0	Current RRi value
${\mathrm{RR}}_{-1}$	Previous RRi value
${\mathrm{RR}}_{+1}$	Next RRi value
RR0/avgRR	Current RRi value divided by the average of the previous 42 RRi values
tRR0	(Current RRi value − average RRi)/standard deviation of RRi
${\mathrm{RR}}_{-1}/\mathrm{avgRR}$	Previous RRi value divided by the average RRi
${\mathrm{RR}}_{-1}/\mathrm{RR}0$	Previous RRi value divided by the current RRi value
${\mathrm{RR}}_{+1}/\mathrm{avgRR}$	Next RRi value divided by the average RRi

**Table 2:** Distribution of AAMI heartbeat classes and extracted features for training dataset.

AAMI	MIT-BIH	RRI Features	Morphology and Wavelet Features
Normal (N)	N, L, R, e, j	87,689	90,102
Supraventricular Ectopic Beat (S)	A, a, J, S	2719	2781
Ventricular Ectopic Beat (V)	V, E	6596	7008
Fusion (F)	F	794	802
Unknown (Q)	/, f, Q	8	15

**Table 3:** LSTM.

Layer (Type)	Output Shape	Param #
lstm_1 (LSTM)	(None, 256)	264,192
dense_2 (Dense)	(None, 128)	32,896
dense_3 (Dense)	(None, 5)	645

**Table 4:** BI-LSTM.

Layer (Type)	Output Shape	Param #
bidirectional (Bidirectional)	(None, 512)	528,384
dense (Dense)	(None, 256)	131,328
dense_1 (Dense)	(None, 5)	1285

**Table 5:** Model Summary LSTM FCN.

Layer (Type)	Output Shape	Param #	Connected to
input_layer_2 (InputLayer)	(None, 100, 1)	0	-
permute_1 (Permute)	(None, 1, 100)	0	input_layer_2[0][0]
conv1d_3 (Conv1D)	(None, 1, 128)	102,528	permute_1[0][0]
batch_normalization_3 (BatchNormalization)	(None, 1, 128)	512	conv1d_3[0][0]
activation_3 (Activation)	(None, 1, 128)	0	batch_normalization_3[0][0]
conv1d_4 (Conv1D)	(None, 1, 256)	164,096	activation_3[0][0]
batch_normalization_4 (BatchNormalization)	(None, 1, 256)	1,024	conv1d_4[0][0]
activation_4 (Activation)	(None, 1, 256)	0	batch_normalization_4[0][0]
conv1d_5 (Conv1D)	(None, 1, 128)	98,432	activation_4[0][0]
batch_normalization_5 (BatchNormalization)	(None, 1, 128)	512	conv1d_5[0][0]
lstm_2 (LSTM)	(None, 8)	320	input_layer_2[0][0]
activation_5 (Activation)	(None, 1, 128)	0	batch_normalization_5[0][0]
dropout_1 (Dropout)	(None, 8)	0	lstm_2[0][0]
global_average_pooling1d_1 (GlobalAveragePooling1D)	(None, 128)	0	activation_5[0][0]
concatenate_1 (Concatenate)	(None, 136)	0	dropout_1[0][0], global_average_pooling1d_1[0][0]
dense_3 (Dense)	(None, 5)	685	concatenate_1[0][0]

**Table 6:** Accuracy, precision, recall, and F1-score results of LSTM, Bi-LSTM, and LSTM-FCN models using RRI features.

Model	Accuracy	Precision	Recall	F1-Score
LSTM-Sequential	0.965	0.963	0.965	0.962
Bi-LSTM	0.963	0.962	0.963	0.962
LSTM-FCN	0.963	0.960	0.963	0.960

**Table 7:** Class-wise accuracy results of LSTM, Bi-LSTM, and LSTM-FCN models using RRI features.

Model	N	S	V	F	Q
LSTM-Sequential	0.98	0.73	0.82	0.21	0.00
Bi-LSTM	0.99	0.68	0.80	0.21	0.00
LSTM-FCN	0.98	0.72	0.83	0.22	0.00

**Table 8:** Accuracy, precision, recall, and F1-score results of LSTM, Bi-LSTM, and LSTM-FCN models using morphology features.

Model	Accuracy	Precision	Recall	F1-Score
LSTM-Sequential	0.987	0.987	0.987	0.987
Bi-LSTM	0.988	0.987	0.988	0.987
LSTM-FCN	0.985	0.985	0.985	0.985

**Table 9:** Class-wise accuracy results of LSTM, Bi-LSTM, and LSTM-FCN models using morphology features.

Model	N	S	V	F	Q
LSTM-Sequential	0.99	0.82	0.96	0.84	0.00
Bi-LSTM	0.99	0.88	0.96	0.70	0.20
LSTM-FCN	0.99	0.87	0.97	0.84	0.20

**Table 10:** Accuracy, precision, recall, and F1-score results of LSTM, Bi-LSTM, and LSTM-FCN models using wavelet features.

Model	Accuracy	Precision	Recall	F1-Score
LSTM-Sequential	0.990	0.990	0.990	0.990
Bi-LSTM	0.987	0.987	0.987	0.987
LSTM-FCN	0.990	0.980	0.990	0.980

**Table 11:** Class-wise accuracy results of LSTM, Bi-LSTM, and LSTM-FCN models using Wavelet features.

Model	N	S	V	F	Q
LSTM-Sequential	0.99	0.84	0.96	0.77	0.20
Bi-LSTM	0.99	0.86	0.96	0.74	0.20
LSTM-FCN	0.99	0.87	0.97	0.75	0.20

**Table 12:** Jetson Orin Nano developer kit specification [26].

Feature	Specification
CPU	6-core Arm Cortex-A78AE v8.2 64-bit
GPU	NVIDIA Ampere, 1024 CUDA Cores, 32 Tensor Cores
AI Performance	Up to 40 TOPS (67 TOPS with Super)
Memory	8 GB LPDDR5, 68 GB/s bandwidth
Storage	microSD slot, M.2 NVMe slots
Power Consumption	7 W–15 W–25 W modes
Connectivity	USB-C, USB 3.2 (x4), DisplayPort, Ethernet, Wi-Fi, Bluetooth
Software	Ubuntu 22.04, JetPack R36.3.0

## Data Availability

For training the classifiers, we use a dataset from the MIT-BIH Arrhythmia Database (https://physionet.org/content/mitdb/1.0.0/, accessed 5 November 2024). The experimental data are available from the corresponding authors on reasonable request.
